# Supplementary material for: Mechanism of action of non-camptothecin inhibitor Genz-644282 in topoisomerase I inhibition
Source: Commun Biol. 2022 Sep 16;5:982. doi: 10.1038/s42003-022-03920-w (PMC9481636; doi:10.1038/s42003-022-03920-w)
Supplement: Supplementary file 2 — Supplementary Information [file 42003_2022_3920_MOESM2_ESM.pdf]

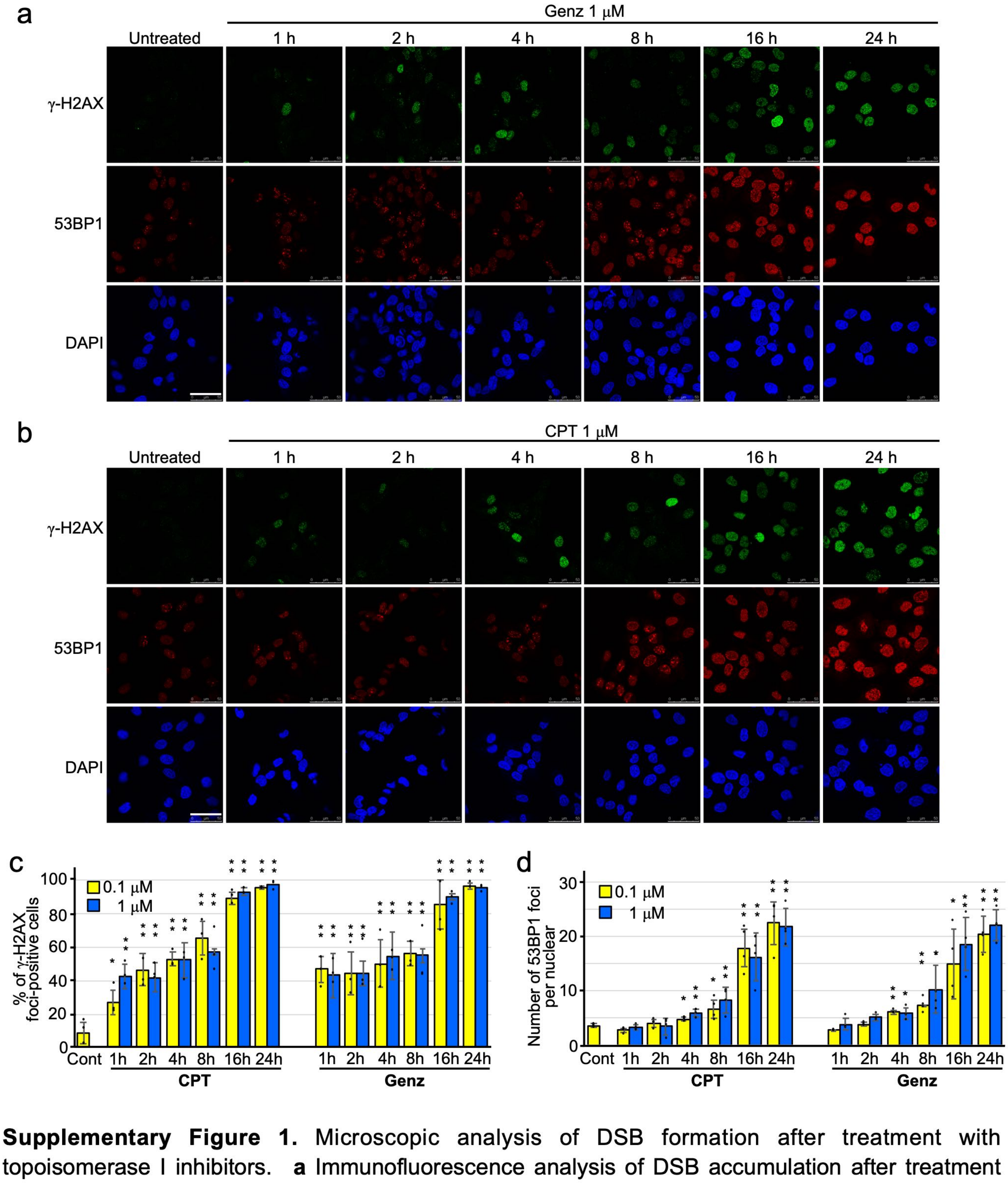

Supplementary Figure 1. Microscopic analysis of DSB formation after treatment with topoisomerase I inhibitors. **a** Immunofluorescence analysis of DSB accumulation after treatment with Genz by detecting foci of the DSB markers -H2AX and 53BP1. Scale bar represents 50 μm. **b** Immunofluorescence analysis of DSB accumulation after treatment with CPT by detecting foci of the DSB markers γ-H2AX and 53BP1. Scale bar represents 50 μm. **c** Quantification of cells positive for γ-H2AX foci. The means and standard deviations were determined from three independent experiments. **d** Quantification of large 53BP1 foci in the nucleus on one confocal plain. The means and standard deviations were determined from four independent experiments.

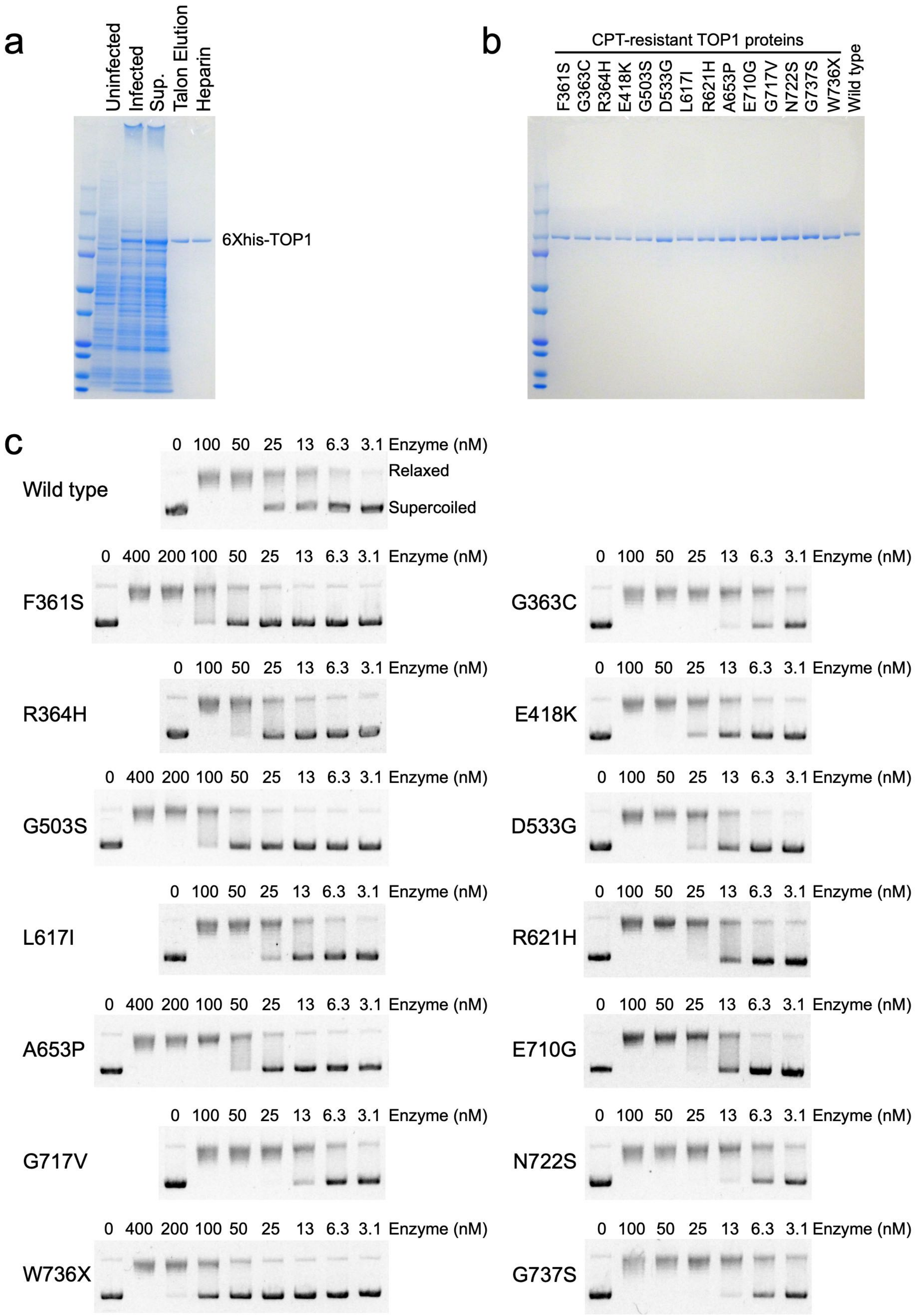

Supplementary Figure 2. Purification of CPT-resistant TOP1 and confirmation of its catalytic activities. **a** SDS-PAGE of purified recombinant His-tagged TOP1. **b** SDS-PAGE of purified wild-type and CPT-resistant TOP1 proteins, such as F361S, G363C, R364H, E418K, G503S, D533G, L617I, R621H, A653P, E710G, G717V, N722S, W736X, and G737S mutants. **c** Confirmation of the DNA relaxation activities of CPT-resistant TOP1 proteins by agarose gel electrophoresis.

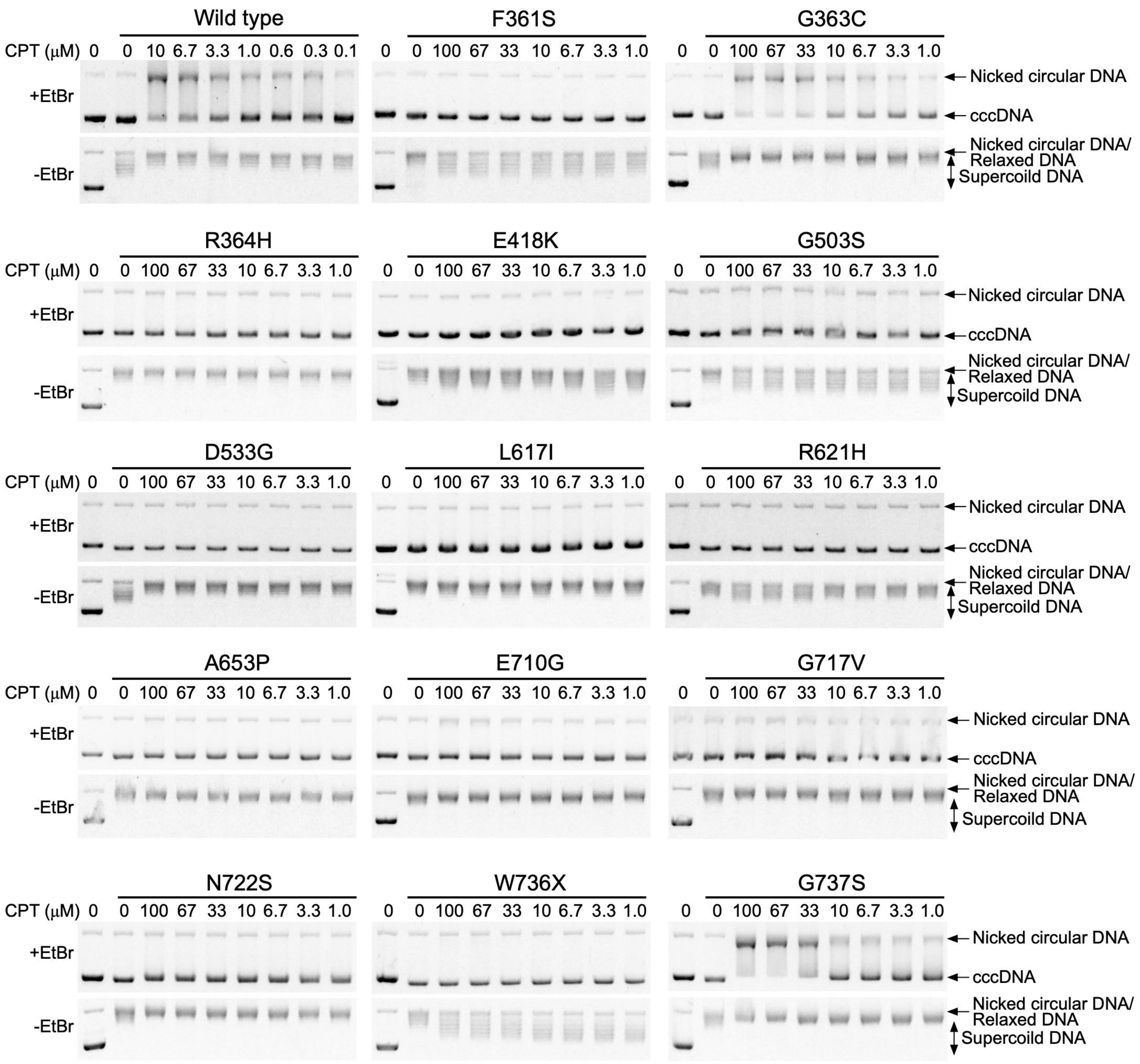

Supplementary Figure 3. Confirmation of the CPT resistance of CPT-resistant TOP1 proteins. Relaxation and nicking activities of the wild-type and various TOP1-mutated proteins were analysed by agarose gel electrophoresis in the presence of CPT.

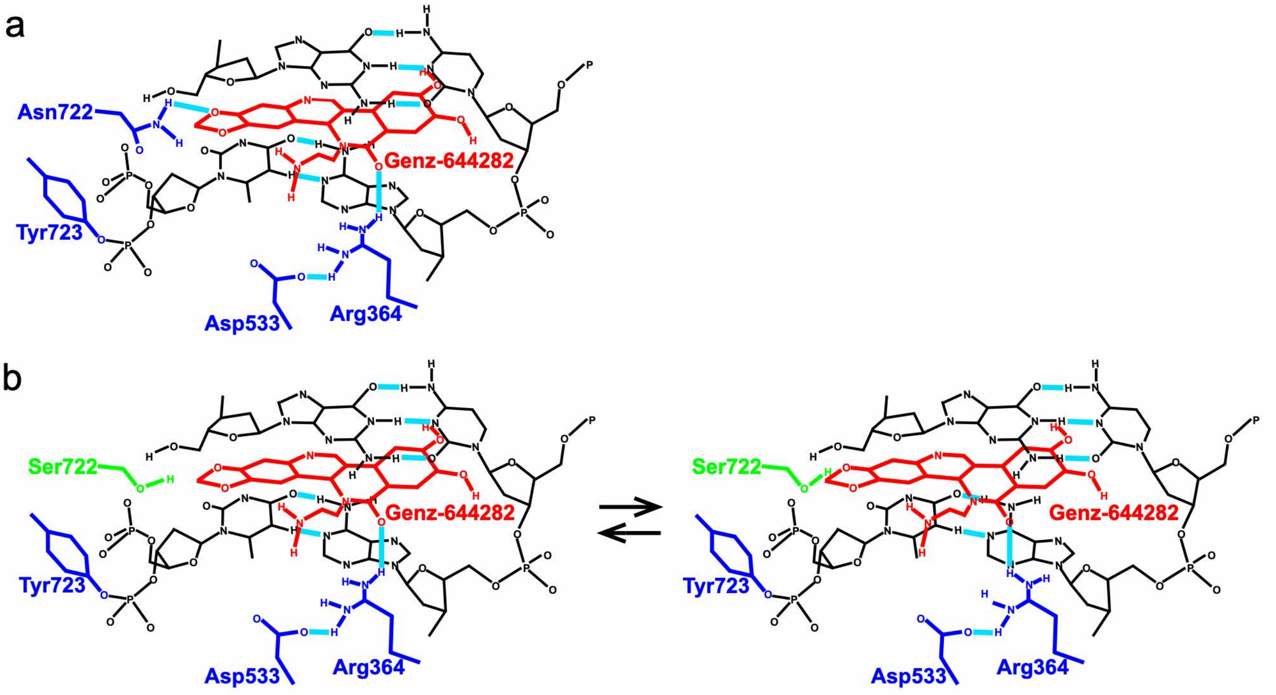

Supplementary Figure 4. Alternative model. **a** Action of Genz on the wild-type TOP1. The direct interaction between asparagine 722 and CPT inhibits the re-ligation of cleaved DNA.
b Action of Genz on CPT-resistant TOP1 carrying N722S mutation. Substitutions of asparagine residue 722 for amino acids with small side chains, such as serine and alanine, provided sufficient space to allow deep invasion of Genz into the active site, which resulted in strong inhibition of the re-ligation step. The structure of the DNA is represented by black lines and letters. Residues of TOP1 protein are represented by blue and green lines and letters. Inhibitors, CPT and Genz, are represented by red lines and letters. Hydrogen bonds between TOP1 and the inhibitor are represented by light blue lines. Additional factors, such as water and phosphate, are represented by purple lines and letters.

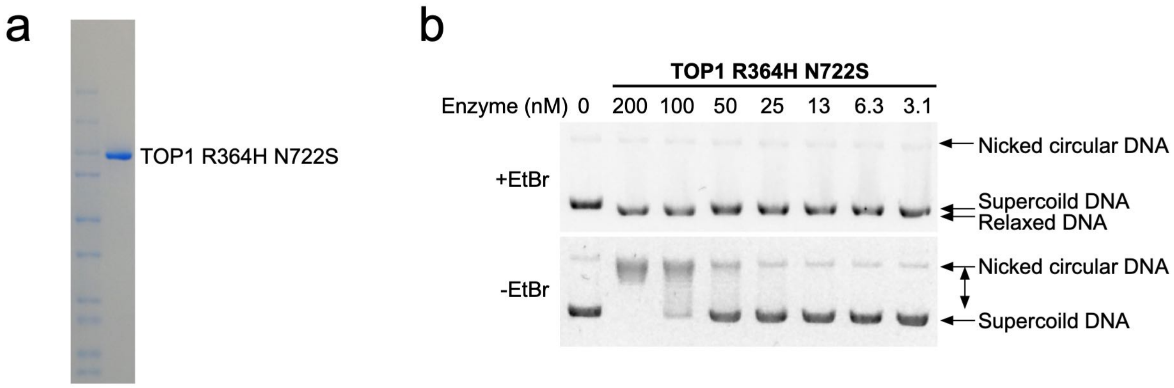

Supplementary Figure 5. Purification of R364H N722S double-mutant TOP1 and confirmation of its catalytic activities. **a** SDS-PAGE of purified R364H N722S double-mutant TOP1. **b** Confirmation of DNA relaxation activities of R364H N722S double-mutant TOP1.

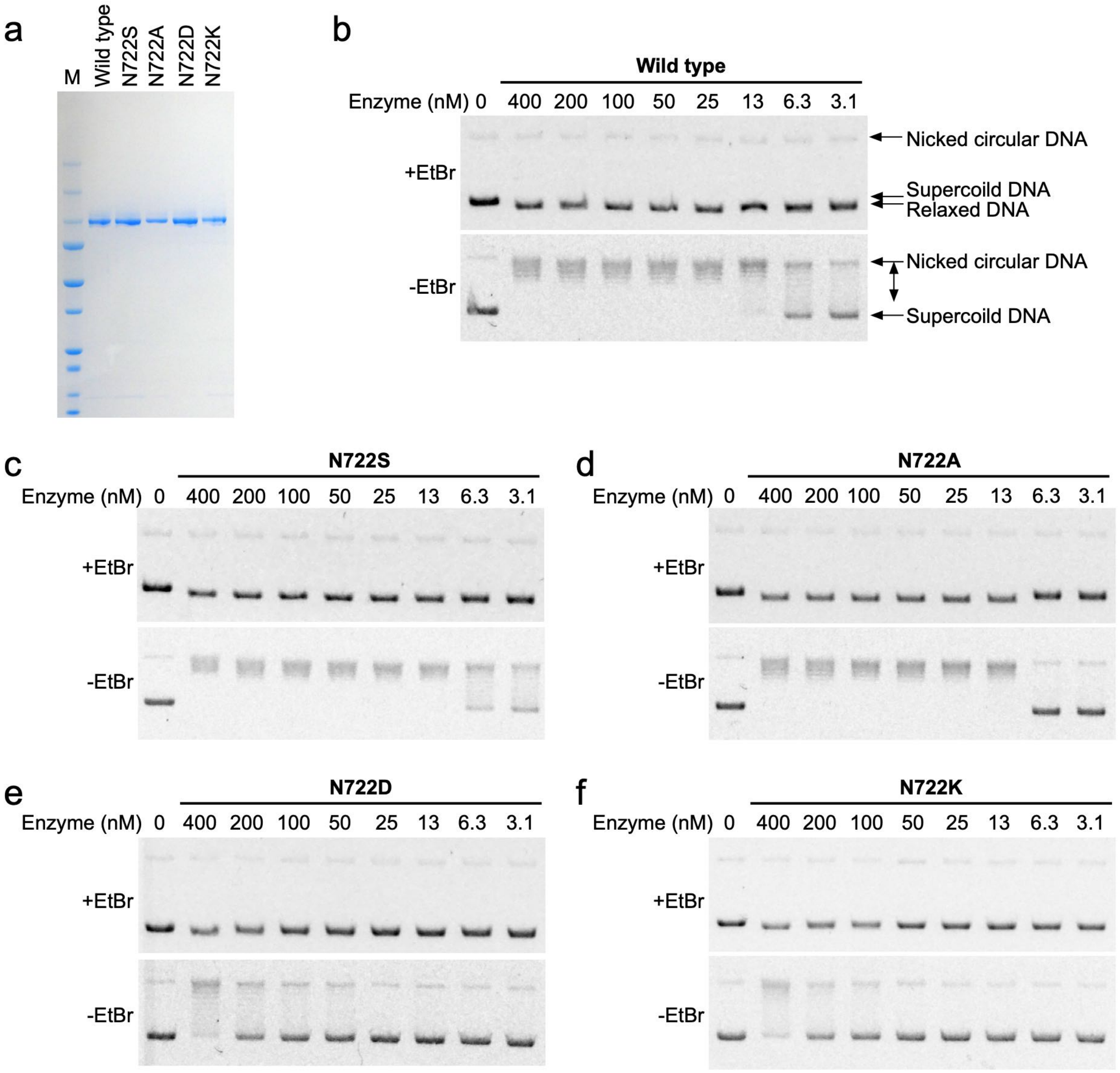

Supplementary Figure 6. Purification of CPT-resistant TOP1 and confirmation of its catalytic activities. **a** SDS-PAGE of purified recombinant His-tagged TOP1 and CPT-resistant TOP1 proteins. **b** Confirmation of DNA relaxation activities of wild-type TOP1. **c–f** Confirmation of DNA catalytic activity of CPT-resistant TOP1 protein. Relaxation activities of CPT-resistant TOP1 proteins carrying N722S mutation **(c)**, N722A mutation **(d)**, N722D mutation **(e)**, and N722K mutation **(f)** were analysed by agarose gel electrophoresis.
