# Supplementary material for: Mechanism of action of non-camptothecin inhibitor Genz-644282 in topoisomerase I inhibition
Source: Commun Biol. 2022 Sep 16;5:982. doi: 10.1038/s42003-022-03920-w (PMC9481636; doi:10.1038/s42003-022-03920-w)
Supplement: Supplementary file 3 — Description of Additional Supplementary Files [file 42003_2022_3920_MOESM3_ESM.pdf]

Description of Additional Supplementary Files

File name: Supplementary Data 1
Description: The source data behind the graphs in the paper.

File name: Supplementary Data 2
Description: Uncropped original gel images in the paper.
