# Supplementary material for: Mechanism of action of non-camptothecin inhibitor Genz-644282 in topoisomerase I inhibition
Source: Commun Biol. 2022 Sep 16;5:982. doi: 10.1038/s42003-022-03920-w (PMC9481636; doi:10.1038/s42003-022-03920-w)

Uncropped original files

Fig 2c

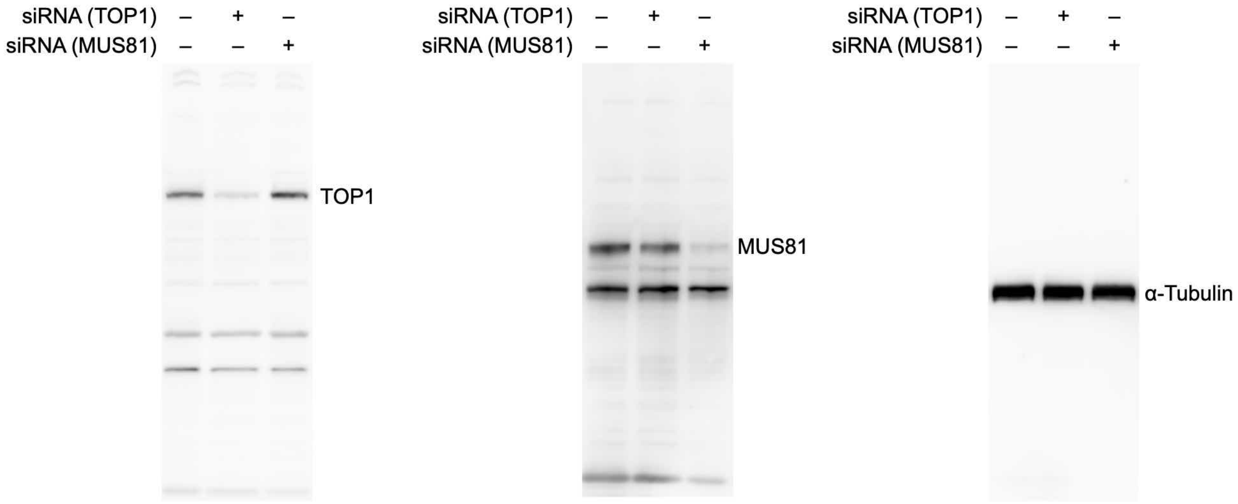

Uncropped original files

Fig 3a

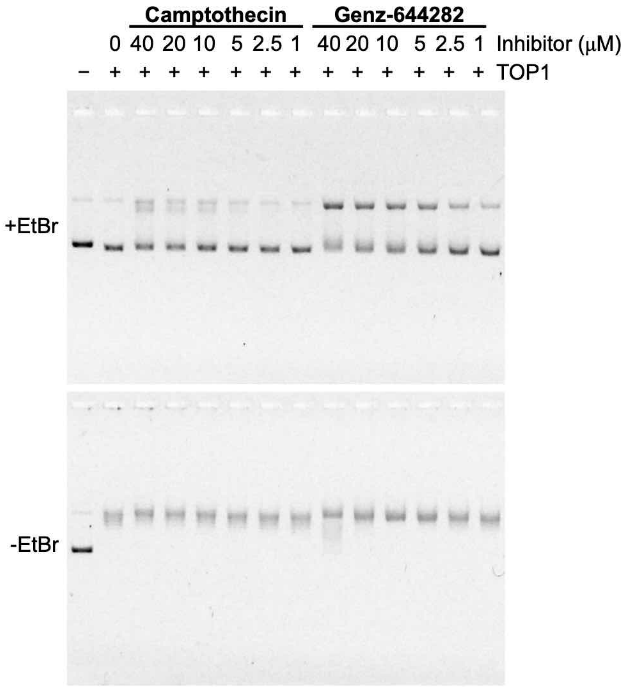

Fig 3b

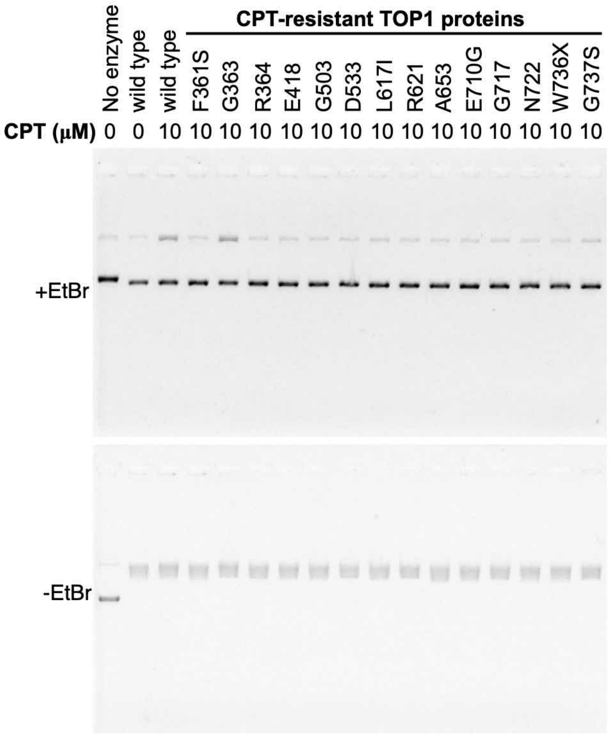

FIg 3c

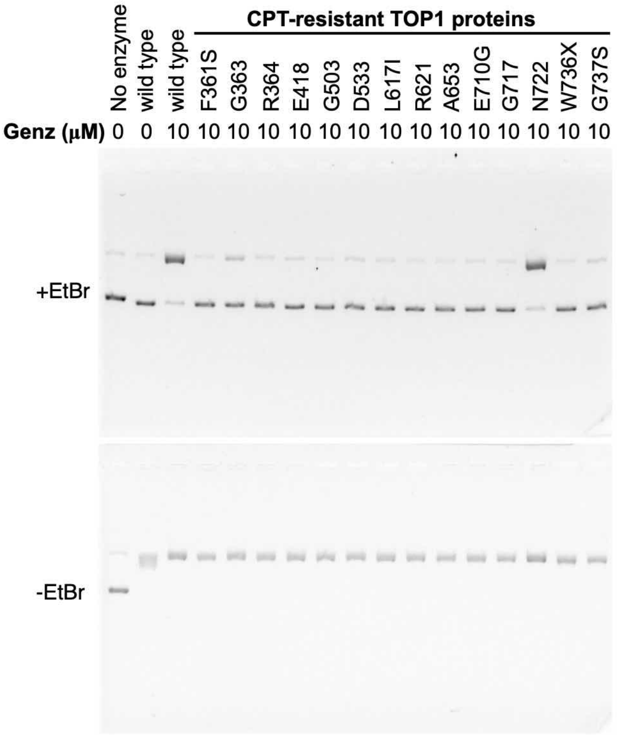

Uncropped original files

Fig 7a

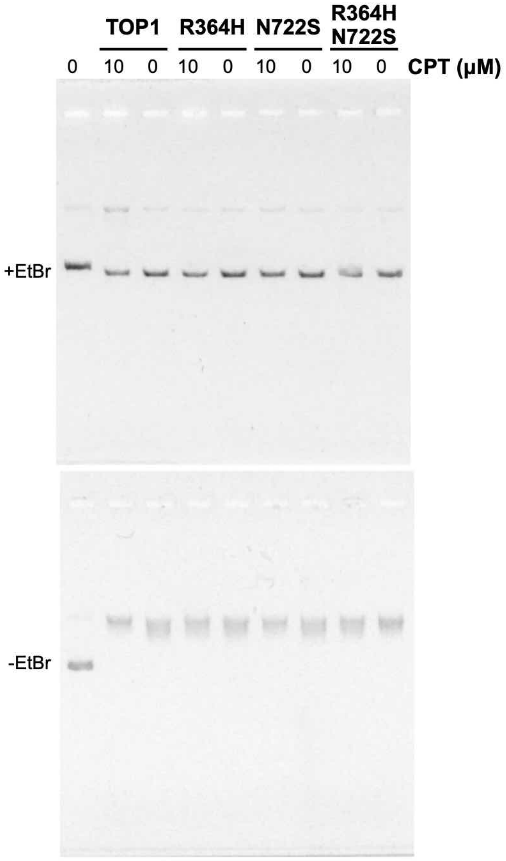

Fig 7c

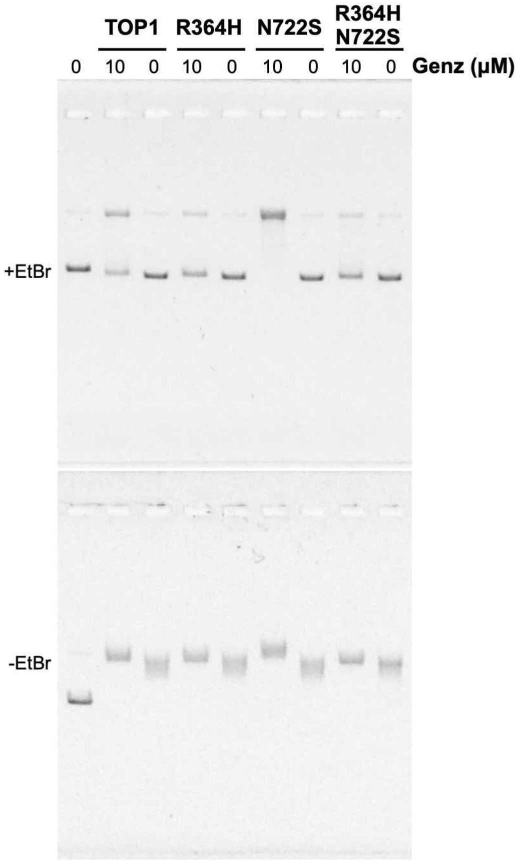

Uncropped original files

Fig 8a

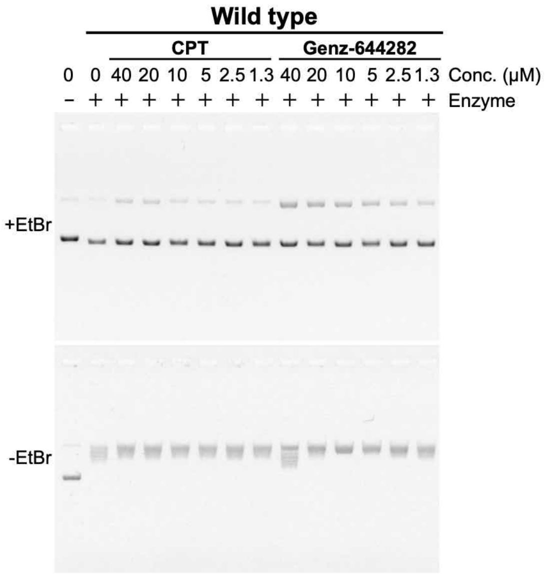

Fig 8b

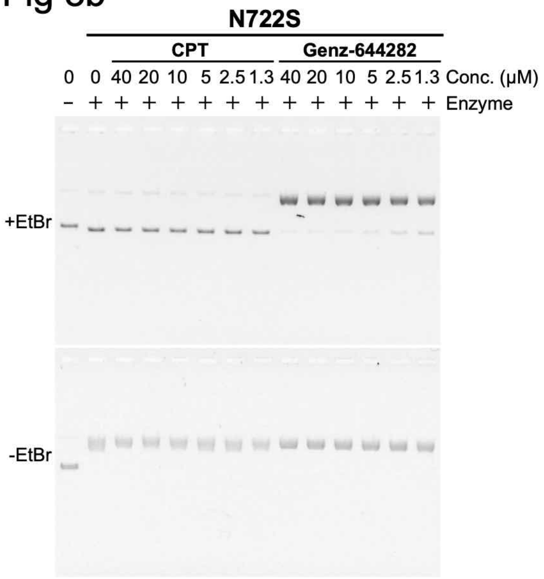

Fig 8c

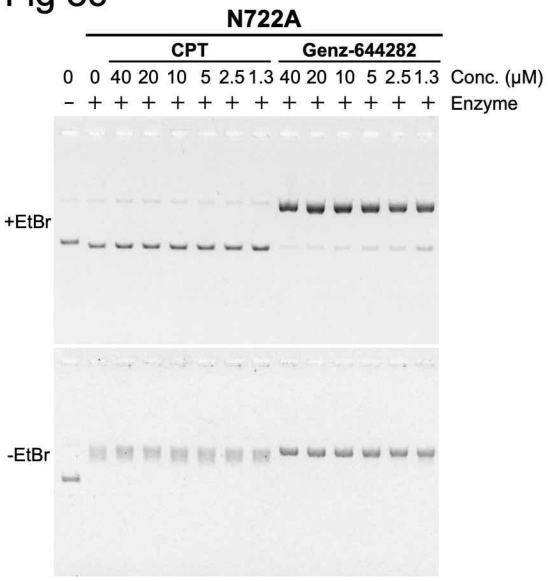

Fig 8d

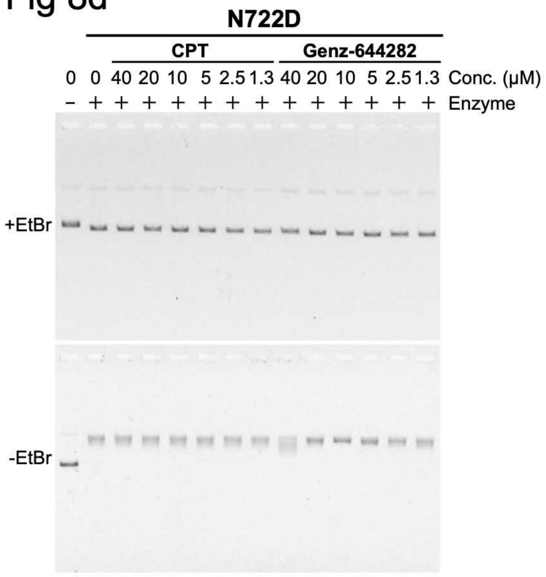

Fig 8e

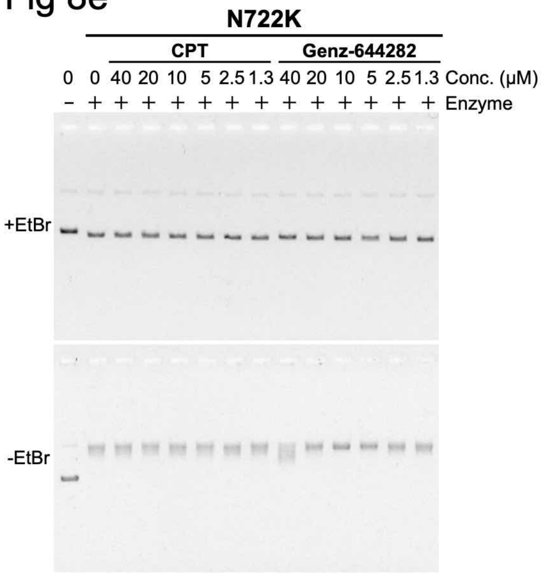

Uncropped original files

Suppementary Fig 2

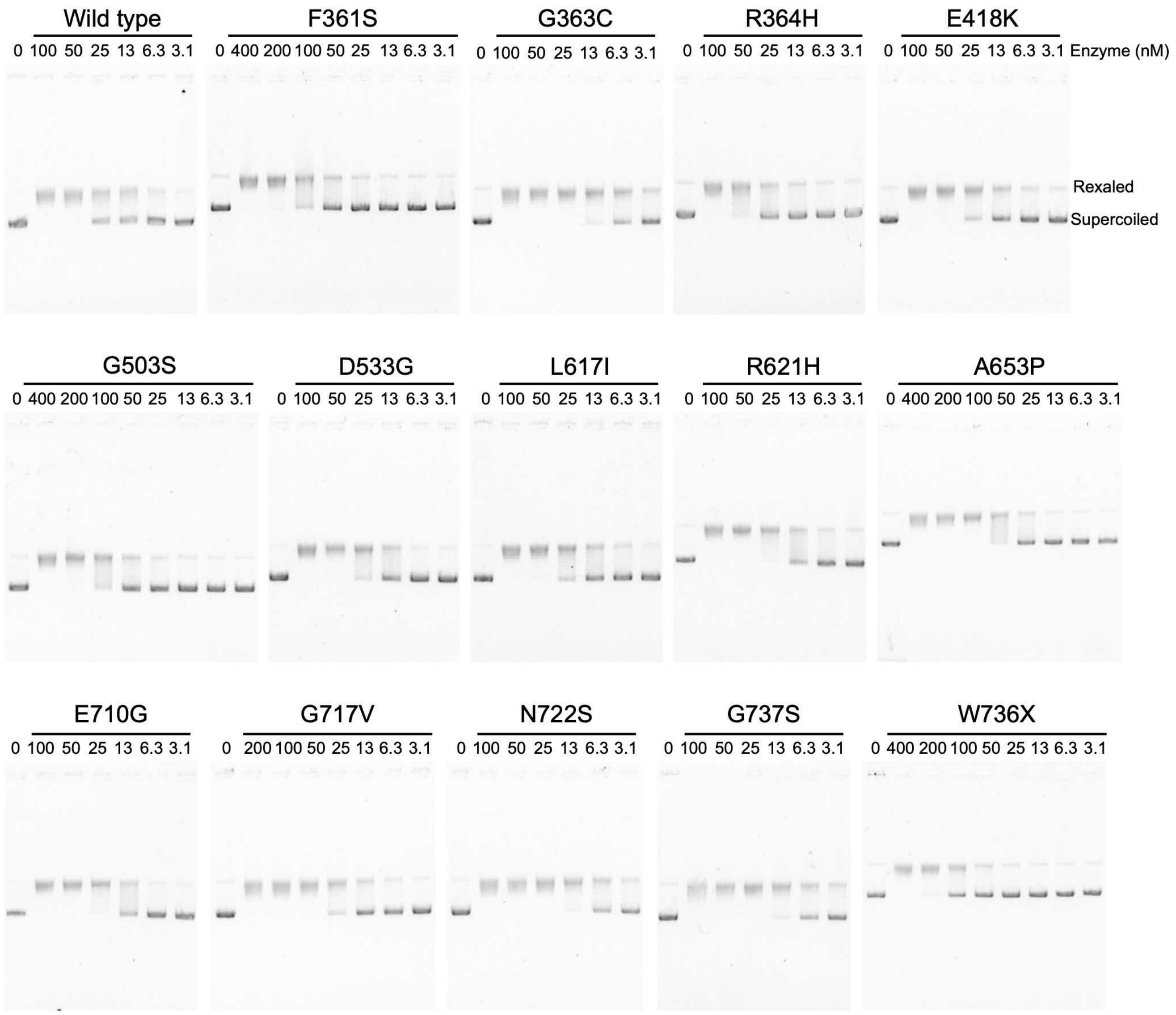

Uncropped original files

Suppementary Fig 3.

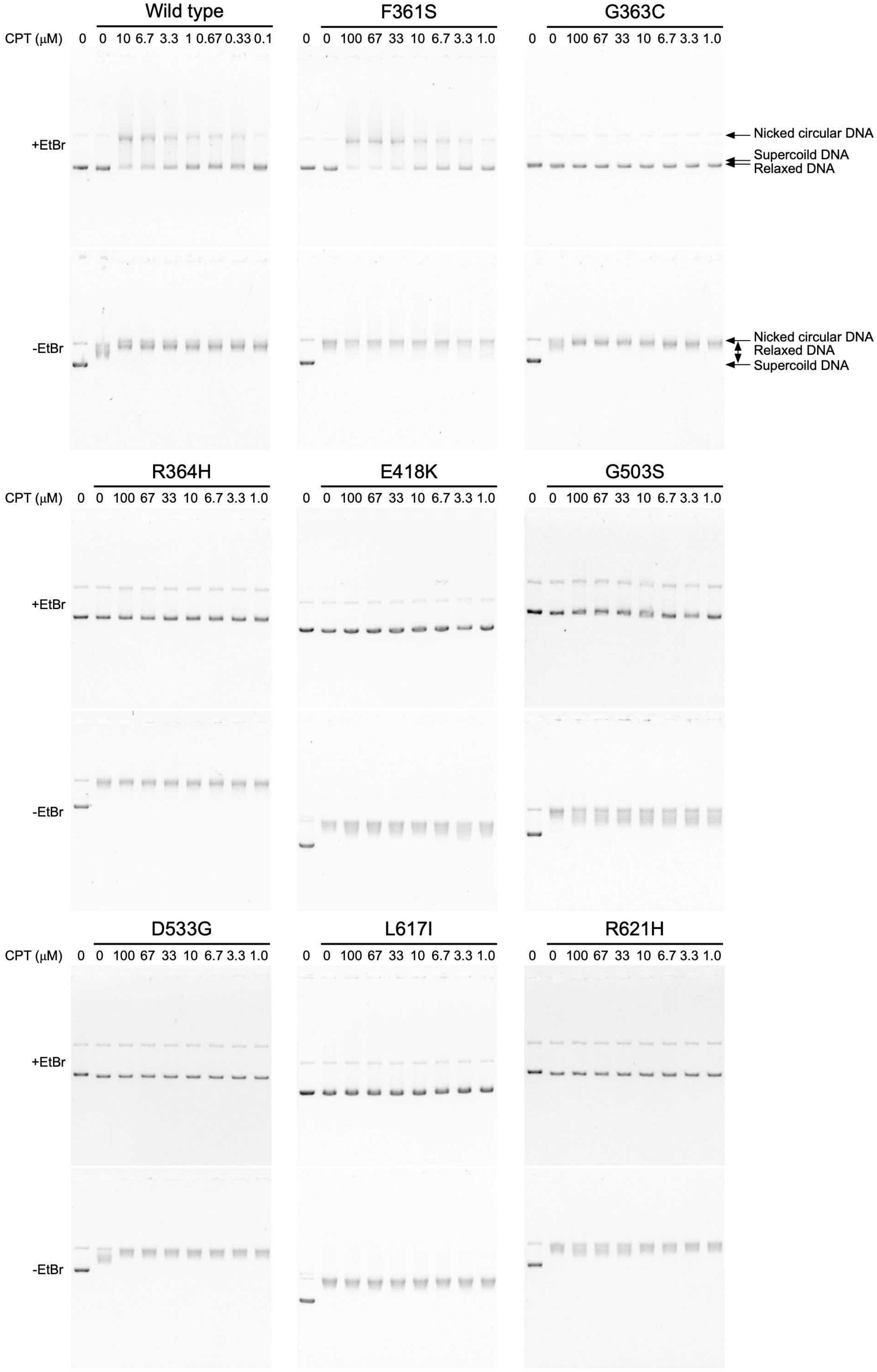

Uncropped original files

Suppementary Fig 3.

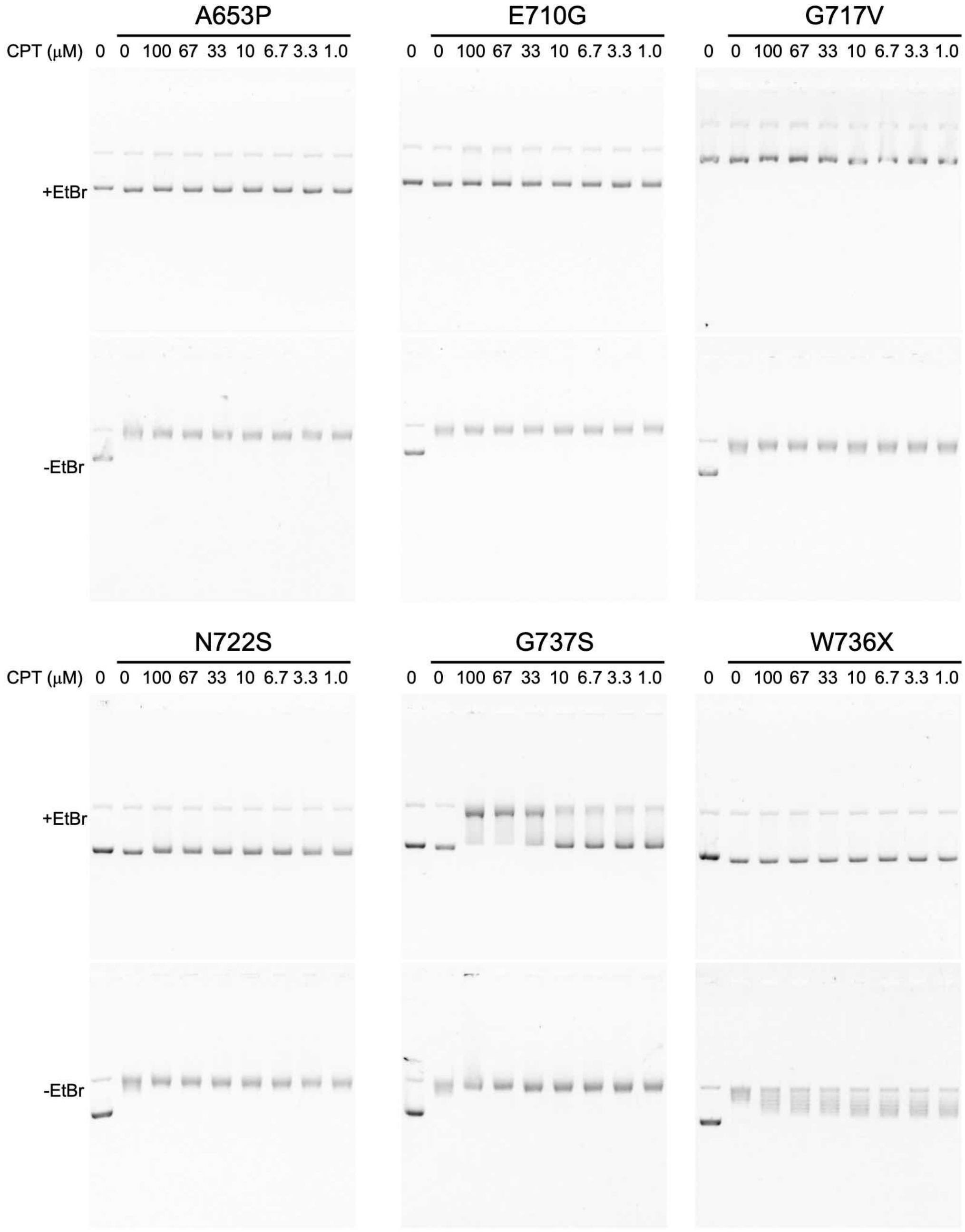

Uncropped original files

Suppementary Fig 5b

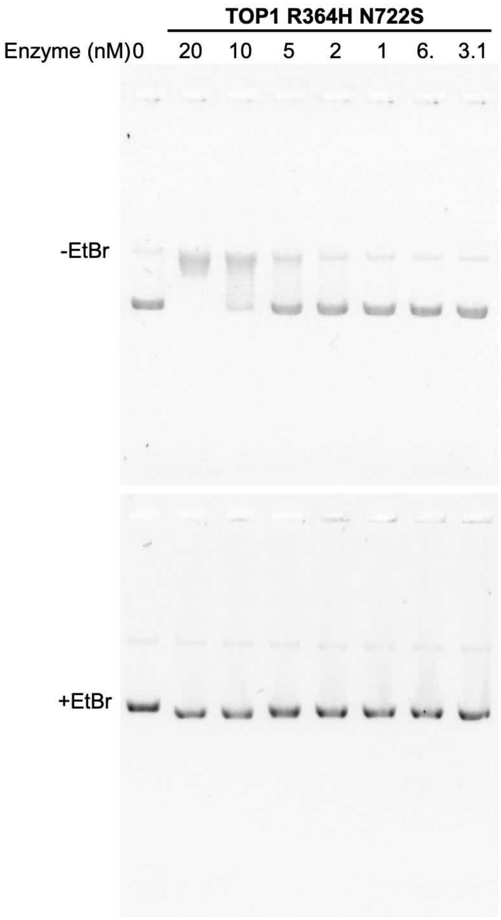

Uncropped original files

Supplementary Fig 6

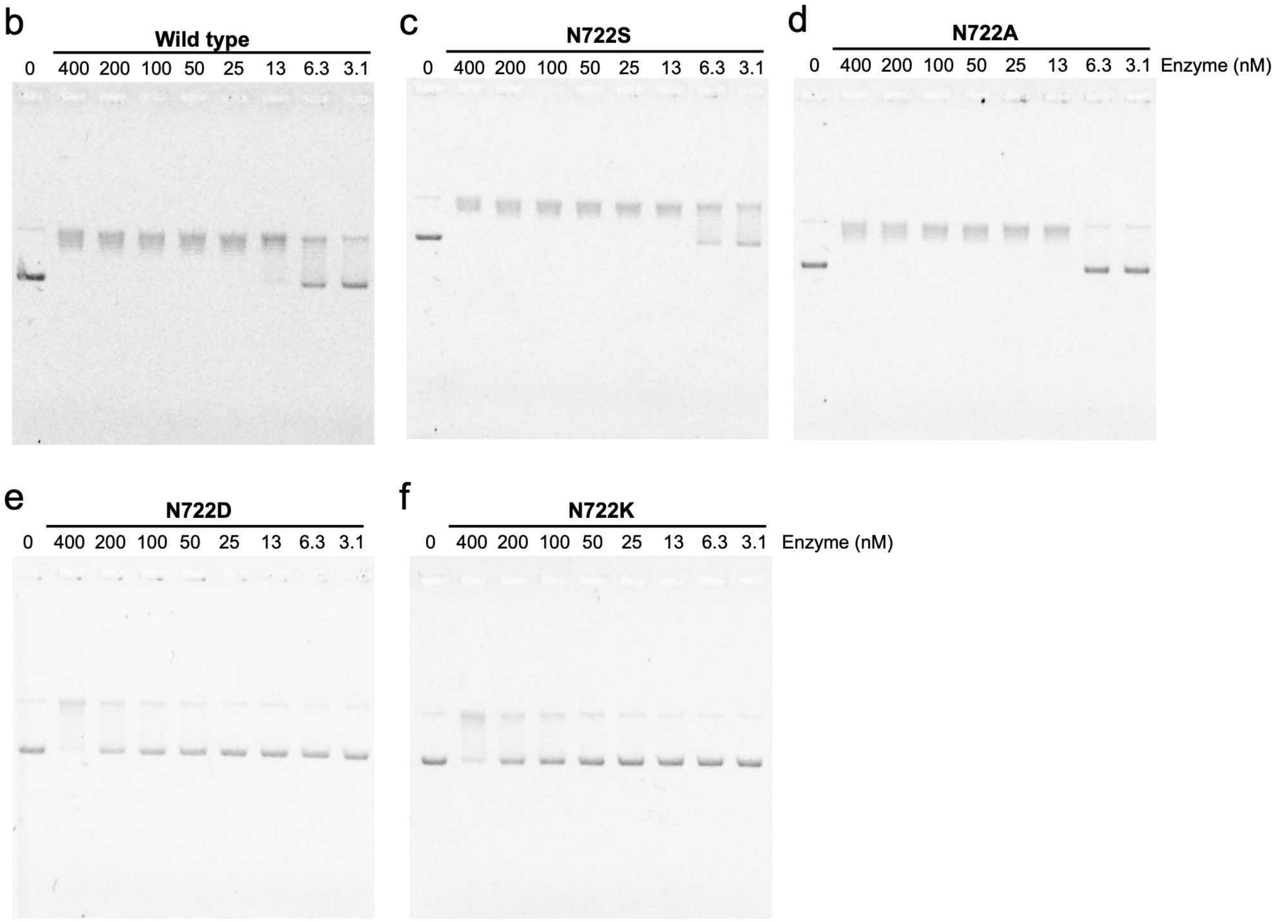

Supplement: Supplementary file 5 — Supplementary Data 2. [file 42003_2022_3920_MOESM5_ESM.pdf]
