# Supplementary material for: Mechanism of action of non-camptothecin inhibitor Genz-644282 in topoisomerase I inhibition
Source: Commun Biol. 2022 Sep 16;5:982. doi: 10.1038/s42003-022-03920-w (PMC9481636; doi:10.1038/s42003-022-03920-w)
Supplement: Supplementary file 6 — Reporting summary [file 42003_2022_3920_MOESM6_ESM.pdf]

nature portfolio

Corresponding author(s): Katsuhiro Hanada, Yoshihiro Nishida

Last updated by author(s): Aug 9, 2022

Reporting Summary

Nature Portfolio wishes to improve the reproducibility of the work that we publish. This form provides structure for consistency and transparency in reporting. For further information on Nature Portfolio policies, see our Editorial Policies and the Editorial Policy Checklist.

Statistics

For all statistical analyses, confirm that the following items are present in the figure legend, table legend, main text, or Methods section.

n/a	Confirmed	
☐	☒	The exact sample size (n) for each experimental group/condition, given as a discrete number and unit of measurement
☐	☒	A statement on whether measurements were taken from distinct samples or whether the same sample was measured repeatedly
☐	☒	The statistical test(s) used AND whether they are one- or two-sided<br>*Only common tests should be described solely by name; describe more complex techniques in the Methods section.*
☐	☒	A description of all covariates tested
☐	☒	A description of any assumptions or corrections, such as tests of normality and adjustment for multiple comparisons
☐	☒	A full description of the statistical parameters including central tendency (e.g. means) or other basic estimates (e.g. regression coefficient) AND variation (e.g. standard deviation) or associated estimates of uncertainty (e.g. confidence intervals)
☐	☒	For null hypothesis testing, the test statistic (e.g. F, t, r) with confidence intervals, effect sizes, degrees of freedom and P value noted<br>*Give P values as exact values whenever suitable.*
☒	☐	For Bayesian analysis, information on the choice of priors and Markov chain Monte Carlo settings
☒	☐	For hierarchical and complex designs, identification of the appropriate level for tests and full reporting of outcomes
☒	☐	Estimates of effect sizes (e.g. Cohen's d, Pearson's r), indicating how they were calculated

Our web collection on statistics for biologists contains articles on many of the points above.

Software and code

Policy information about availability of computer code

Data collection	Leica TCS SP8 STED, Analytik Jena Rotaphor, GE Typhoon FLA7000, GE Las-4000 mini, Thermo Scientific Multi skan Sky.
Data analysis	GE Image Quant TL, Avogadro software 1.2.0, Autodock Vina software 1.1.2, PyMol Molecular Graphics System (Schrodinger, LLC).

For manuscripts utilizing custom algorithms or software that are central to the research but not yet described in published literature, software must be made available to editors and reviewers. We strongly encourage code deposition in a community repository (e.g. GitHub). See the Nature Portfolio guidelines for submitting code & software for further information.

Data

Policy information about availability of data

All manuscripts must include a data availability statement. This statement should provide the following information, where applicable:

- Accession codes, unique identifiers, or web links for publicly available datasets
- A description of any restrictions on data availability
- For clinical datasets or third party data, please ensure that the statement adheres to our policy

Provide your data availability statement here.

Human research participants

Policy information about studies involving human research participants and Sex and Gender in Research.

Reporting on sex and gender	*Use the terms sex (biological attribute) and gender (shaped by social and cultural circumstances) carefully in order to avoid confusing both terms. Indicate if findings apply to only one sex or gender; describe whether sex and gender were considered in study design whether sex and/or gender was determined based on self-reporting or assigned and methods used. Provide in the source data disaggregated sex and gender data where this information has been collected, and consent has been obtained for sharing of individual-level data; provide overall numbers in this Reporting Summary. Please state if this information has not been collected. Report sex- and gender-based analyses where performed, justify reasons for lack of sex- and gender-based analysis.*
Population characteristics	*Describe the covariate-relevant population characteristics of the human research participants (e.g. age, genotypic information, past and current diagnosis and treatment categories). If you filled out the behavioural & social sciences study design questions and have nothing to add here, write "See above."*
Recruitment	*Describe how participants were recruited. Outline any potential self-selection bias or other biases that may be present and how these are likely to impact results.*
Ethics oversight	*Identify the organization(s) that approved the study protocol.*

Note that full information on the approval of the study protocol must also be provided in the manuscript.

Field-specific reporting

Please select the one below that is the best fit for your research. If you are not sure, read the appropriate sections before making your selection.

☒ Life sciences ☐ Behavioural & social sciences ☐ Ecological, evolutionary & environmental sciences

For a reference copy of the document with all sections, see nature.com/documents/nr-reporting-summary-flat.pdf

Life sciences study design

All studies must disclose on these points even when the disclosure is negative.

Sample size	Means and SD of survival curves were obtained from triplicated experiments. Means and SD of PFGE experiments were obtained from for independent experiments.
Data exclusions	There was no exclusions for data analysis.
Replication	All experiments were reproductive.
Randomization	Fibroblast cells was randomly selected form healthy population. CPT-resistant cells and their control cells were selected from public sources. CPT-resistant TOP1 mutations were randomly selected from previous studies (Tsurutani, J. et al. Point mutations in the topoisomerase I gene in patients with non-small cell lung cancer treated with irinotecan. Lung Cancer 35, 299-304 (2002). Bailly, C. et al. The camptothecin-resistant topoisomerase I mutant F361S is cross-resistant to antitumor rebeccamycin derivatives. A model for topoisomerase I inhibition by indolocarbazoles. Biochemistry 38, 8605-8611 (1999). Benedetti, P., Fiorani, P., Capuani, L. & Wang, J.C. Camptothecin resistance from a single mutation changing glycine 363 of human DNA topoisomerase I to cysteine. Cancer Res 53, 4343-4348 (1993). Fiorani, P. et al. Single mutation in the linker domain confers protein flexibility and camptothecin resistance to human topoisomerase I. J Biol Chem 278, 43268-43275 (2003). Urasaki, Y. et al. Characterization of a novel topoisomerase I mutation from a camptothecin-resistant human prostate cancer cell line. Cancer Res 61, 1964-1969 (2001). Chang, J.Y., Liu, J.F., Juang, S.H., Liu, T.W. & Chen, L.T. Novel mutation of topoisomerase I in rendering cells resistant to camptothecin. Cancer Res 62, 3716-3721 (2002). Pan, P., Li, Y., Yu, H., Sun, H. & Hou, T. Molecular principle of topotecan resistance by topoisomerase I mutations through molecular modeling approaches. J Chem Inf Model 53, 997-1006 (2013). Urasaki, Y., Takebayashi, Y. & Pommier, Y. Activity of a novel camptothecin analogue, homocamptothecin, in camptothecin-resistant cell lines with topoisomerase I alterations. Cancer Res 60, 6577-6580 (2000). Urasaki, Y. et al. Use of camptothecin-resistant mammalian cell lines to evaluate the role of topoisomerase I in the antiproliferative activity of the indolocarbazole, NB-506, and its topoisomerase I binding site. Cancer Res 61, 504-508 (2001). Gongora, C. et al. New Topoisomerase I mutations are associated with resistance to camptothecin. Mol Cancer 10, 64 (2011). Tesauro, C. et al. Molecular mechanism of the camptothecin resistance of Glu710Gly topoisomerase IB mutant analyzed in vitro and in silico. Mol Cancer 12, 100 (2013). Wang, L.F. et al. Identification of mutations at DNA topoisomerase I responsible for camptothecin resistance. Cancer Res 57, 1516-1522 (1997)).
Blinding	No blind test was applied.

Reporting for specific materials, systems and methods

We require information from authors about some types of materials, experimental systems and methods used in many studies. Here, indicate whether each material, system or method listed is relevant to your study. If you are not sure if a list item applies to your research, read the appropriate section before selecting a response.

Materials & experimental systems

n/a	Involved in the study
☐	☒ Antibodies
☐	☒ Eukaryotic cell lines
☒	☐ Palaeontology and archaeology
☒	☐ Animals and other organisms
☒	☐ Clinical data
☒	☐ Dual use research of concern

Methods

n/a	Involved in the study
☒	☐ ChIP-seq
☒	☐ Flow cytometry
☒	☐ MRI-based neuroimaging

Antibodies

Antibodies used

mouse anti-MUS81 antibody (MTA30 2G10/3) (1:10,000, ab-14387; Abcam),
mouse anti-Topo I antibody (C-21) (1:10,000, sc-32736; Santa Cruz Biotechnology),
mouse anti-?-tubulin antibody (10G10) (1:10,000, 017-25031; Fujifilm)

Validation

mouse anti-MUS81 antibody (MTA30 2G10/3) (1:10,000, ab-14387; Abcam) https://www.abcam.co.jp/mus81-antibody-mta30-2g103-ab14387.html
mouse anti-Topo I antibody (C-21) (1:10,000, sc-32736; Santa Cruz Biotechnology) https://www.scbt.com/p/topo-i-antibody-c-21?productCanUrl=topo-i-antibody-c-21&_requestid=284877
mouse anti-?-tubulin antibody (10G10) (1:10,000, 017-25031; Fujifilm) https://labchem-wako.fujifilm.com/jp/product/detail/W01W0101-2503.html

Eukaryotic cell lines

Policy information about cell lines and Sex and Gender in Research

Cell line source(s)

The MRC5sv cell line was kindly provided by Dr. Roland Kanaar. The RPMI8402 cell line was kindly provided by Dr. Yoshiaki Ohnishi. CCRF-CEM (ATCC CCL-119), and CEM/C2 (ATCC CRL-2264) were purchased from American Type Culture Collection (ATCC) (Manassas, VA, USA). CPT-K5 was purchased the Japanese Collection of Research Bioresources (JCRB) Cell Bank (Osaka, Japan).

Authentication

Cells were originally obtained from public sources. Therefore, none of these were authenticated.

Mycoplasma contamination

Because mycoplasma were not detected by DAPI staining, we judged all cells were not contaminated with mycoplasma.

Commonly misidentified lines
(See ICLAC register)

None
